# GELT: A graph embeddings based lite-transformer for knowledge tracing

**DOI:** 10.1371/journal.pone.0301714

**Published:** 2024-05-07

**Authors:** Zhijie Liang, Ruixia Wu, Zhao Liang, Juan Yang, Ling Wang, Jianyu Su

**Affiliations:** 1 School of Computer Science, Sichuan Normal University, Chengdu, Sichuan, China; 2 School of Marxism, Sichuan Water Conservancy Vocational College, Chengdu, Sichuan, China; 3 Network and Information Center, Southwest Petroleum University, Chengdu, Sichuan, China; Zhejiang University of Technology, CHINA

## Abstract

The development of intelligent education has led to the emergence of knowledge tracing as a fundamental task in the learning process. Traditionally, the knowledge state of each student has been determined by assessing their performance in previous learning activities. In recent years, Deep Learning approaches have shown promising results in capturing complex representations of human learning activities. However, the interpretability of these models is often compromised due to the end-to-end training strategy they employ. To address this challenge, we draw inspiration from advancements in graph neural networks and propose a novel model called GELT (Graph Embeddings based Lite-Transformer). The purpose of this model is to uncover and understand the relationships between skills and questions. Additionally, we introduce an energy-saving attention mechanism for predicting knowledge states that is both simple and effective. This approach maintains high prediction accuracy while significantly reducing computational costs compared to conventional attention mechanisms. Extensive experimental results demonstrate the superior performance of our proposed model compared to other state-of-the-art baselines on three publicly available real-world datasets for knowledge tracking.

## 1. Introduction

With the widespread popularity of large-scale online platforms [1], knowledge tracing (KT) has emerged as a central research focus in intelligent tutoring systems [2]. knowledge tracing is a fundamental and crucial task to support adaptive learning [3] services, which aims to predict whether students will answer questions correctly on subsequent steps, based on their historical learning records. However, due to the wide range of learning activities and the complexity of the knowledge domain, achieving accurate knowledge state diagnosis remains a challenging task.

From the perspective of artificial intelligence algorithms, a knowledge tracking task can be defined as a supervised sequential learning problem [4]. Given a student’s participated past exercise interactions *Q* = {*q*_1_, *q*_2_, …, *q*_*t*_}, where *q*_1−*t*_ represents a set of discrete time indices, we can formulate the task using a generic model where the student’s knowledge and performance are characterized as follows:

$$\begin{array}{c} \begin{array}{cc} & e_{t+1}∼g(e_{t})\\ & r_{t+1}∼f(e_{t+1}) \end{array} \end{array}$$ (1)

In the above formulations, *e*_*t*_ represents the student’s current knowledge level, which is not directly observed. The function *f*() describes how the student’s knowledge influences their responses, and the function *g*() captures the evolution of the student’s knowledge over time.

The goal of knowledge tracing is to predict whether the student will have the ability to correctly answer the exercise question *e*_*t*+1_ at the next time step *t* + 1. In other words, the task aims to predict *P*(*r*_*t*+1_|*e*_*t*+1_, *W*_*t*_), where *W*_*t*_ denotes the knowledge state at time *t*. The variable *r*_*t*+1_ predicts some aspect of the student’s next interaction, with 1 indicating a correct feedback and 0 signifying an incorrect response.

Over the past three decades, researchers have made significant progress in addressing the Knowledge Tracing problem. Several methods have been developed with three prominent models being widely utilized: Bayesian Knowledge Tracing [5] (BKT), Additive Factor Model [6] (AFM), and Deep Knowledge Tracing [7] (DKT).

The BKT method regards the underlying knowledge state as an explicit binary variable. After each interactive learning session, the binary variable’s value is updated using a Hidden Markov Model (HMM) [8], depending on whether the student has achieved mastery of a specific skill. However, the BKT method faces limitations in capturing the relationships between different skill points. This is primarily due to the ambiguous correspondence between real-world skill points and variables. Consequently, BKT lacks the capability to effectively model complex knowledge.

The AFM method assumes that learning occurs as a gradual process of change. This method utilizes two variables, specifically the number of practice sessions and the learning rate, to indicate their influence on the student’s cognitive state. It facilitates the transition from static evaluation to dynamic analysis of the student’s cognitive states. However, the AFM method heavily depends on expert experience for annotating domain knowledge, leading to substantial manual costs.

The Deep Knowledge Tracing method serves as a valuable tool in predicting the student’s knowledge state by employing recurrent neural networks (RNN) [9]. This method transforms the input learning state into vector representations through the utilization of one-hot encoding [10] or compression perception algorithms [11]. By conducting feature extraction in the input and hidden layers, the output layer successfully predicts the student’s cognitive states on the subsequent question. Differentiated from conventional machine learning approaches, recurrent neural networks possess the advantage of effectively representing temporal states with high-order and continuity, making them more suited for modeling complex knowledge systems. DKT surpasses traditional machine learning methods in terms of prediction performance. However, a limitation of DKT lies in the lack of interpretability in the parameters of its hidden layers. For instance, there may be variations in difficulty levels among questions that share the same skill points.

**Fig 1:**
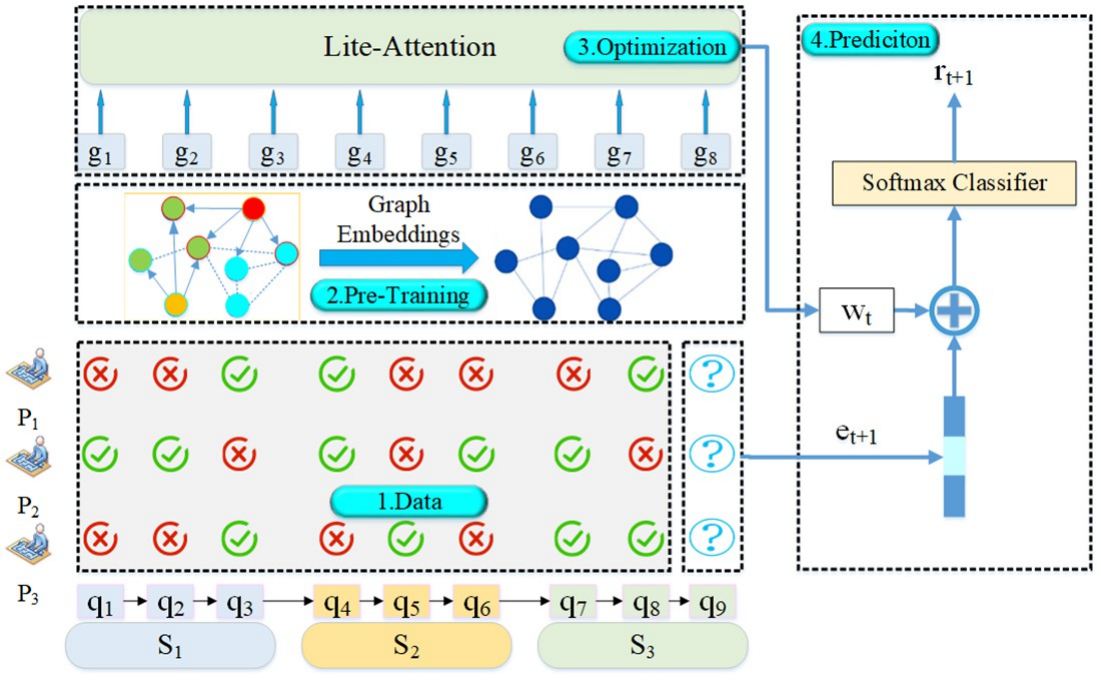
Overview of the GELT method.

As shown in Fig 1, the symbol {*S*_1_…*S*_n_} corresponds to skills, {*q*_1_…*q*_*m*_} represents the questions, and denotes the students. Each student completes a set of questions that form a sequence, with markings of “correct” and “incorrect”. It is evident that each skill can be associated with multiple questions, and a question can correspond to multiple skills. Consequently, certain prior studies [12, 13] have incorporated question attributes as an additional factor to complement skill inputs. Drawing inspiration from the effectiveness of pre-training embeddings [14] via a bipartite graph framework, our approach employs a graph convolutional network [15] to aggregate embeddings for both skills and questions, taking into account high-order relations. Specifically, explicit question-skill relations capture direct associations between questions and skills, while implicit question similarity incorporates similarities between different questions to provide additional context during the tracing process. Additionally, skill similarity accounts for similarities between different skills. By acquiring these pretrained embeddings, we can directly utilize them as input features for KT models, resulting in a substantial enhancement of accuracy.

In addition to input features, knowledge state prediction is a crucial aspect of KT. The Transformer model [16], incorporating an attention mechanism [17], has gained popularity in recent years for its ability to greatly enhance prediction accuracy in deep learning models. However, the high training costs and computational requirements of the Transformer model present limitations in its application to knowledge tracking tasks. To overcome this limitation, we propose Lite-Transformer, an energy-saving attention mechanism that is both simple and effective. By leveraging kernelized hashing [18], Lite-Transformer maps queries and keys into compact binary codes, resulting in an attention mechanism with linear complexity. This approach allows us to replace floating-point multiplications in attention with simple additions, leading to significant reductions in training computational costs.

To summarize, we proposes Graph Embeddings based Lite-Transformer for the knowledge tracing task. The primary contributions of this work are outlined below:

1. Utilizing graph convolutional networks to aggregate question and skill embeddings, allowing for the effective capture of complex relationships between questions and skills.
2. Introducing a simple yet effective energy-saving attention mechanism for predicting knowledge state. This approach maintains high prediction accuracy while significantly reducing computational costs compared to the conventional attention mechanism.
3. By conducting extensive experiments on three knowledge tracing benchmark datasets, this study provides compelling evidence that our proposed approach, GELT, surpasses state-of-the-art baselines in terms of performance.

The remaining sections of this paper are organized as follows. Section 2 provides related works on knowledge tracing and recent developments in applying deep learning to knowledge state prediction. Section 3 presents the details of our method. Section 4 presents the results of our experimental analysis and provides remarks. Finally, in Section 5, the paper concludes by summarizing the work and highlighting potential future innovations.

## 2. Relate works

Knowledge tracing has gained significant prominence in the field of educational data mining in recent years. According to different modeling approaches, existing knowledge tracing models can be categorized into two distinct groups: traditional machine learning based methods and deep learning based methods.

### 2.1 Machine learning based methods

Traditional machine learning KT methods mainly involve two types: Bayesian Knowledge Tracing [19] based on statistics and Additive Factor Model [20] based on logistic regression.

In the 1994, Bayesian Knowledge Tracing was proposed as a probabilistic model [21] to capture the dependencies between concepts and mastery levels. The mastery of each knowledge point by the student is represented as a binary variable *P*(*K*_*t*_) ∈ {0, 1}, which can take values of either 0 or 1. Each variable indicates whether the student has achieved mastery in a specific knowledge point *K*_*t*_. Subsequently, a Hidden Markov Model [8] is utilized to monitor the fluctuations in the student’s knowledge state and predict the probability of the student achieving mastery in the corresponding knowledge point.

The prediction outcomes generated by the standard BKT method exhibit statistical significance and offer a satisfactory level of interpretability [22]. Nevertheless, the modeling process of BKT relies on three assumptions, which possess certain limitations [23]: The student’s response sequence is restricted to a single knowledge point, thus hindering the analysis of response sequences encompassing multiple knowledge points; The absence of a forgetting phenomenon in the student’s learning process is assumed, which may oversimplify the actual learning dynamics; Both the student’s performance and knowledge mastery state are treated as binary variables, which fails to capture the intricate nature of the learning process. Unfortunately, these assumptions fail to comprehensively capture and describe the intricate nature of the human learning process.

To overcome the aforementioned limitations of the BKT method, a novel approach called the Additive Factor Model has been proposed, which combines traditional machine learning techniques with psychometric theory [24]. Unlike BKT, the AFM assumes that learning is a gradual process rather than an abrupt transition. Instead of estimating the student’s latent knowledge state, the AFM directly predicts the probability of the student providing correct answers to practice questions. Moreover, numerous researchers have extended the standard AFM from various perspectives, including incorporating parameter constraints [25], introducing error factors [26], and emotional attitudes [27]. However, these methods only take into account the historical interactions of each questions or skills independently, without effectively capture the correlations between questions and skills.

### 2.2 Deep learning based methods

The rapid emergence of deep learning [28] has garnered extensive attention from researchers due to its remarkable feature extraction capability. Consequently, many researchers have adopted deep learning techniques in the domain of Knowledge Tracing. One notable example is Deep Knowledge Tracing [7], which utilizes a recurrent neural network to accurately capture the temporal and semantic features of exercise texts. Moreover, it significantly outperforms the traditional BKT method, demonstrating a 20% increase in the AUC (Area Under Curve) value.

The Dynamic Key-Value Memory Network (DKVMN) [29] utilizes a deep network to automatically detect the relationship between questions and their associated underlying skills, while simultaneously tracking the state of each concept.

Through the aforementioned comparison, it becomes evident that the DKT model encounters the challenge of non-interpretable [30]. parameter updates caused by the utilization of deep neural networks. In contrast, the DKVMN model is more interpretable as it explicitly represents the updates of the skill representation matrix (keys) and the knowledge state representation matrix (values).

In Knowledge Tracing tasks, there are often various relational structures present, including the similarity between skills, dependencies among skills, and the resemblance between questions and their corresponding skills. In order to effectively tackle the knowledge tracing problem and capture the inherent relational structures, there has been a recent trend to leverage the capabilities of graph representation learning strategies, such as Graph Neural Networks (GNNs) [31].

The Graph-based knowledge tracing (GKT) model [12] constructs a similarity graph of skills in a random manner. Subsequently, it autonomously aggregates the weights of the graph nodes to aid in prediction. Several research works have employed the convolutional approach for analyzing and processing graph-structured data. Yang et al. proposed a framework that utilizes Graph Convolutional Networks (GCNs) [13] to incorporate high-order question-skill relational graph structures into representation tasks for complex concepts. This framework is further applied to knowledge tracing tasks, effectively enhancing the interpretability of parameters.

The Pre-training Embeddings via Bipartite Graph (PEBG) [14] method was introduced to enhance the accuracy of knowledge tracing by obtaining pre-trained exercise embeddings. To incorporate all of these relations into skill-question embeddings, the authors construct a bipartite graph that includes these features along with skill difficulties. Subsequently, these features are integrated within the defined bipartite graph to generate the pre-trained exercise embeddings. Based on their experiments, the researchers found that the exercise embeddings obtained through their approach have led to significant improvements in the performance of KT models, including models like DKT.

The Transformer architecture [16] represents a novel neural architecture that leverages the attention mechanism to encode input source data into robust and informative features. Over the past year, the emergence of the Transformer model has led to enhanced accuracy in various computer vision (CV) [32] and natural language processing (NLP) [33] tasks. Inspired by the successful adoption of the Transformer architecture, several studies have been conducted to integrate an attention mechanism into knowledge tracing models.

Self-Attention Knowledge Tracing (SAKT) [34] is a pioneering model in the field of knowledge tracing that integrates attention mechanisms. Notably, SAKT utilizes multiple attention heads [17] to capture the representations of attention matrices. The attention matrix contains relative weights for various representative sub-spaces, which indicate the importance of predicting a student’s response to the current question. Ultimately, SAKT merges these attention matrices from different representative sub-spaces and forecasts a student’s performance using a feed-forward network.

In recent KT research, cutting-edge models utilizing deep neural networks have demonstrated impressive performance. While these models excel in task execution, their limited interpretability often presents obstacles for direct utilization in personalized learning environments. Accordingly, the AKT [35] and FGKT [36] models are investigating the deployment of efficient attention mechanisms to capture unique exercise characteristics and delve into detailed information at the exercise level. Concurrently, these models dynamically incorporate learners’ prior knowledge throughout the knowledge transfer process, thereby enhancing the interpretability of knowledge tracing. This methodology aims to assist learners in achieving exceptional learning outcomes and a personalized learning experience.

The paper integrates two influential approaches: the interpretability of the GNN network in modeling the relationship between skills and questions, and the robust modeling capability of the Transformer model in capturing features. The primary objective of this study is to investigate the application of a fusion model that improves both the accuracy and interpretability of knowledge tracing tasks.

## 3. The proposed method

In this section, we will provide an in-depth overview of the GELT model used for knowledge tracing. We will delve into the training process of the graph-based embedding model, as well as the construction of an Lite-Transformer model that focuses on improving accuracy while minimizing computational costs.

### 3.1 Data preprocessing

Knowledge Tracing is a task that aims to predict a student’s performance on future questions by utilizing their past interactions with the learning material. To achieve this, we retrieve sets of answer records from the backend of the online learning system. These records contain valuable information that can be utilized for analysis and predicting the student’s future knowledge acquisition.

Specifically, we extract two main collections: the question information $C_{X}$ and the skill point information $C_{S}$. The number of questions is denoted as *N*_*X*_, while the number of skill points is represented as *N*_*S*_. To facilitate further processing, we encode each question and skill point, resulting in *M* − 1 dimensional coding vectors. The specific dimension *M* of the coding vector can be determined based on the requirements of our analysis. When extracting each answer record, we collect key information including question coding, skill point coding, student ID, and the student answer indicator. The student answer indicator is represented by a binary value, 1 for a correct answer and 0 for an incorrect answer.

By utilizing these answer records, we can create a comprehensive collection of historical behavior records for each student. Each record includes the question ID that the student answered, the corresponding answer indicator, and the set of skill point IDs related to the question. These historical behavior records offer valuable insights into the students’ progress and performance over time.

### 3.2 Embedding representation based on graph structure

To construct the structure for graph embeddings, we utilize *N*_*X*_ questions and *N*_*S*_ skill points as nodes. An edge is created between a question and a skill point if they appear together in the same answer record. This process generates the structure graph *G*, which represents the connections between the questions and skill points. After creating the structure graph *G*, initial vectors are assigned to each node. Then, graph embedding techniques are applied to derive embedding vectors for both the question and skill point nodes. These embedding vectors have a dimension of *M*, resulting in the question feature matrix $X\in R^{N_{X}\times M}$ and the skill point feature matrix $S\in R^{N_{S}\times M}$.

**Fig 2:**
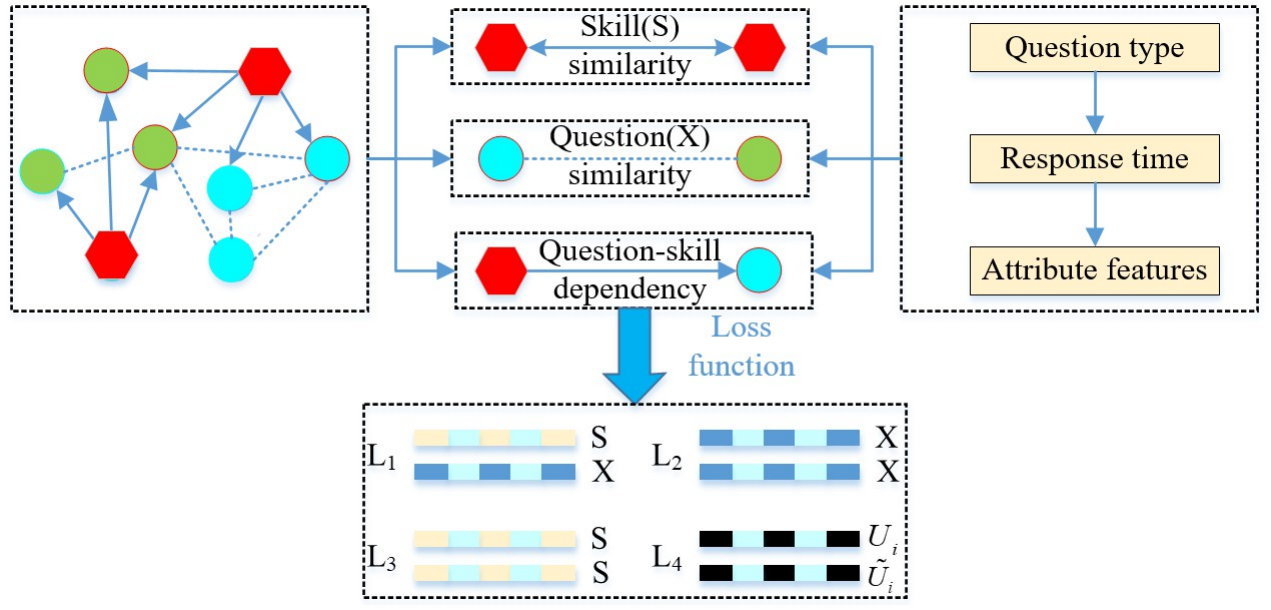
The graph embedding process in GELT. When a student answers a question, GELT follows a series of steps. Firstly, it aggregates the skill features associated with the answered question (L1). Next, it updates the question features related to the skill (L2) and calculates the similarity for each skill node (L3). In addition, the model incorporates the difficulty information of the question as an attribute feature (L4). Lastly, the student’s knowledge states are updated through supervised learning using four loss functions.

As shown in Fig 2, the associated structure graph of questions and skill points includes node attributes, node connection edges, and relationship attributes. During the graph embedding process, neighborhood sampling is conducted to gather the neighbors of each node through aggregation operations. The node information is then updated based on the results of the neighborhood sampling, resulting in high-order embedding vectors for each node.

To enhance the accuracy of the embedding vectors, the loss function for graph embedding in this study integrates the local dependency between questions and skill points, as well as their similarities. Additionally, to extract knowledge effectively from the graph structure, the accuracy of all students’ responses to the current question node is converted into attribute features and embedded into the graph structure. This ensures that the learned graph embedding vectors contain information about the difficulty of the questions.

The formula for calculating the graph embedding loss function is as follows:

$$\begin{array}{c} Loss=\left(1-\alpha \right)*(L_{1}\left(X,S\right)+L_{2}\left(X\right)+L_{3}\left(S\right))+\left(\alpha \right)*L_{4}\left(X,S,\theta \right) \end{array}$$ (2)

where *L*_1_(*X*, *S*) represents the loss function for the dependency between questions and skill points, and its calculation method is as follows:

For the *i*- th question and the *j*- th skill point, where *i* ∈ {1, 2, …, *N*_*X*_} and *j* ∈ {1, 2, …, *N*_*S*_}, let *x*_*i*_ represent the current feature vector of the *i*- th question, and *s*_*j*_ represent the current feature vector of the *j*- th skill point. If there is a connection edge between the *i*- th question and the *j*- th skill point in the graph *G*, the dependency coefficient *r*_*ij*_ = 1; otherwise, *r*_*ij*_ = 0.

The local proximity ${\tilde{r}}_{ij}$ between the *i*- th question and the *j*- th skill point is calculated using the following formula:

$$\begin{array}{c} {\tilde{r}}_{ij}=\sigma (x_{i}^{T}s_{j}) \end{array}$$ (3)

where the superscript *T* indicates the transpose operation, *σ*() represents the *sigmoid* non-linear activation function. This function normalizes the relation value to the range of [0, 1], indicating the local contiguousness between the skill and the question.

The proximity loss function *L*_1_(*X*, *S*) between the questions and skill points is calculated using the following formula:

$$\begin{array}{c} L_{1}\left(X,S\right)=\sum_{i=1}^{N_{X}}\sum_{j=1}^{N_{S}}-[(r_{ij}\;\text{ln}\;{\tilde{r}}_{ij})+(1-r_{ij})\;\text{ln}\;(1-{\tilde{r}}_{ij})] \end{array}$$ (4)

Based on the associated structure graph *G* of the questions and skill points, if two question nodes share a common neighbor skill point node, the relationship information of those neighbors can be combined to represent the similarity between the questions. Therefore, the cross-entropy loss function *L*_2_(*X*) for calculating the similarity between the questions is computed using the following formula:

$$\begin{array}{c} L_{2}\left(X\right)=\sum_{i=1}^{N_{X}}\sum_{i=1}^{N_{X}}-[({r_{ii^{\prime }}}^{X}\;\text{ln}\;{\tilde{r}}_{ii^{\prime }}^{X})+(1-r_{ii^{\prime }}^{X})\;\text{ln}\;(1-{\tilde{r}}_{ii^{\prime }}^{X})] \end{array}$$ (5)

where $r_{ii^{\prime }}^{X}$ represents the true association between the *i*- th question and the *i*′- th question. If both the *i*- th and *i*′- th questions are neighboring nodes of the same skill point node, $r_{ii^{\prime }}^{X}=1$; otherwise, $r_{ii^{\prime }}^{X}=0$. Consequently, the variable ${\tilde{r}}_{ii^{\prime }}^{X}$ represents the estimated correlation value between the *i*- th and *i*′- th questions, which is calculated as ${\tilde{r}}_{ii^{\prime }}^{X}=\sigma \left(x_{i}^{T}x_{i^{\prime }}\right)$.

Similarly to the questions, if two skill nodes both have a common neighboring node, the relationship information of the neighbors can be aggregated as the similarity between the skill nodes. Therefore, the cross-entropy loss function *L*_3_(*S*) is used to calculate the similarity between the questions according to the following formula:

$$\begin{array}{c} L_{3}\left(S\right)=\sum_{j=1}^{N_{S}}\sum_{j^{\prime }=1}^{N_{S}}-[\left(r_{jj^{\prime }}^{S}\;\text{ln}\;{\tilde{r}}_{jj^{\prime }}^{S}\right)+\left(1-r_{jj^{\prime }}^{S}\right)\;\text{ln}\;\left(1-{\tilde{r}}_{jj^{\prime }}^{S}\right)] \end{array}$$ (6)

where $r_{jj^{\prime }}^{S}$ represents the actual association between skill point *j* and skill point *j*′. If both skill point *j* and skill point *j*′ are neighboring nodes of the same topic node, then $r_{jj^{\prime }}^{S}=1$; otherwise, $r_{jj^{\prime }}^{S}=0$. On the other hand, ${\tilde{r}}_{jj^{\prime }}^{S}=\sigma \left(s_{j}^{T}s_{j^{\prime }}\right)$ represents the estimated correlation between skill points, which is evaluated using the sigmoid function.

In order to comprehensively account for the influence of auxiliary features on students’ knowledge states, we have incorporated students’ accuracy information in the graph model during the embedding phase. For the *i*- th question, the accuracy rate *U*_*i*_ is calculated based on the answer records. This accuracy rate is then mapped to the difficulty attribute feature *C*_*i*_ = *w*^*T*^*U*_*i*_ + *b* using a pre-defined non-linear activation function. In this mapping process, the weight term *w* and bias term *b* of the non-linear activation function are utilized.

To address the issue of gradient vanishing during the model training phase, we employ the *Leaky* *Rectified* *linear* *unit* (*LeakyReLu*) activation function to process the difficulty attribute feature *C*_*i*_. The estimated accuracy value ${\tilde{U}}_{i}$ is computed using the following formula:

$$\begin{array}{c} {\tilde{U}}_{i}=Leaky\left(C_{i}\right)=\text{max}\left(0,\left(w^{T}U_{i}+b\right)\right)+Leak*\text{min}\left(0,w^{T}U_{i}+b\right) \end{array}$$ (7)

where *Leak* is a constant term utilized to preserve negative information in the gradient values. If the value of $w_{i}^{T}U_{i}+b_{i}$ is less than or equal to 0, the output is set to 0.

In this case, the utilization of the *LeakyReLu* activation function results in a sparsity output, thereby leading to faster convergence of the model. The difficulty value of the question is calculated using the following loss function.

$$\begin{array}{c} L_{4}\left(X,S\right)=\frac{1}{N_{X}}\sum_{i=1}^{N_{X}}{\left(U_{i}-{\tilde{U}}_{i}\right)}^{2}+\lambda {∥\varpi ∥}_{2}^{2} \end{array}$$ (8)

where, *ϖ* represents the vector formed by the weight *w* and bias term *b* of the non-linear activation layer. ∥∥_2_ denotes the norm of the regularization term, λ is a coefficient that controls the relationship between the empirical error and the regularization, thereby enhancing the model’s generalization performance.

After constructing the four previously mentioned loss functions, we combine them all to create a cooperative optimization framework during the graph embedding process, following Eq (2). The gradient descent algorithm is utilized to optimize the loss function until convergence. The coefficient controls the contributions between graph constraints and attribute features, and its value is optimized through cross-validation [37]. As a result, this process leads to the trained graph structure and the corresponding embedding vectors for each question node and skill node.

### 3.3 Lite-Transformer architecture for knowledge state prediction

**Fig 3:**
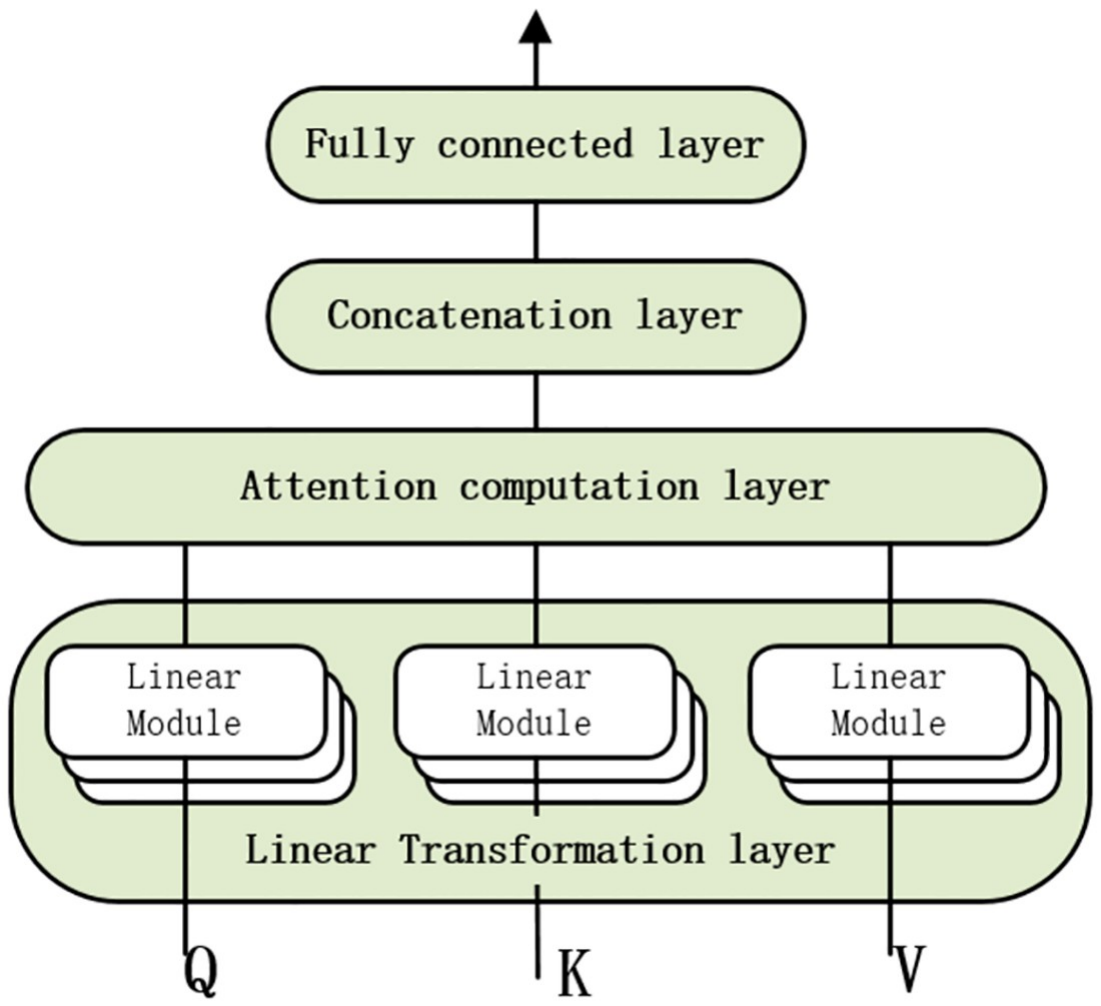
Framework of the attention mechanism.

To accurately predict student’s performance in next step, we have developed a Lite-Transformer model for knowledge state prediction based on the multi-head attention mechanism. As shown in Fig 3, the attention mechanism module comprises the following components: a linear transformation layer is utilized as the convergence module for input features, an attention computation layer for feature selection, a concatenation layer for feature fusion, and a fully connected layer to serve as the final classifier.

#### 1) Linear transformation layer

The linear transformation layer consists of *H* groups of linear modules, where *H* represents the actual number of heads in the multi-head attention mechanism. Each group of linear modules is comprised of three sub-modules, which are responsible for linearly transforming the query *Q*_*d*_, key *K*_*d*_, and value *V*_*d*_ respectively.

After the linear transformation, the input *Q*_*d*_, *K*_*d*_, and *V*_*d*_ matrices are transformed into *Q*_*d*,*h*_ = *Linear*(*Q*_*d*_) = *Q*_*d*_*W*_*Q*,*h*_, *K*_*d*,*h*_ = *Linear*(*K*_*d*_) = *K*_*d*_*W*_*K*,*h*_, and *V*_*d*,*h*_ = *Linear*(*K*_*d*_) = *K*_*d*_*W*_*K*,*h*_, respectively, where $W_{Q,h}\in R^{M\times M}$ represents the weight matrix associated with the *h*- th head.

#### 2) Attention computation layer

For each received *H* groups of weight matrices *Q*_*d*,*h*_, *K*_*d*,*h*_, *V*_*d*,*h*_, the attention computation layer transforms the input features into corresponding attention matrices formulated as follow:

$$\begin{array}{c} \begin{array}{cc} Attention(Q_{d,h},K_{d,h},V_{d,h}) & =\sum_{d=1}^{D}\frac{\text{exp}(\frac{Q_{d,h}\cdot K_{d,h}^{T}}{\sqrt{M}})}{\sum_{h=1}^{H}\text{exp}(\frac{Q_{d,h}\cdot K_{d,h}^{T}}{\sqrt{M}})}V_{h}\\ & \approx \sum_{d=1}^{D}\frac{H\left(Q_{d,h}\right)H{\left(K_{d,h}\right)}^{T}+c}{\sum_{h=1}^{H}H\left(Q_{d,h}\right)H{\left(K_{d,h}\right)}^{T}+c)}V_{d,h}\\ & =\frac{H\left(Q_{d,h}\right)\sum_{h=1}^{H}H\left(K_{d,h}\right)⊗V_{d,h}+\sum_{h=1}^{H}c{V_{d,h}}^{T}}{H\left(Q_{d,h}\right)\sum_{d=1}^{D}H(K_{d,h})+cH} \end{array} \end{array}$$ (9)

where ⊗ represents the outer product operation, the notation *H*() represents the hash function. To avoid division by zero, an offset term *c* is introduced after each inner product operation, ensuring that *H*(*Q*_*d*,*h*_)*H*(*K*_*d*,*h*_)^*T*^ + *c* ≥ 0. Although the above formula reduces the complexity of the attention mechanism to linear operations, it still involves multiple floating-point multiplications. To further improve the efficiency, one possible approach is to perform binary quantization on the queries *Q*_*d*,*h*_ and keys *K*_*d*,*h*_.

Randomly extract *M* sub-matrices $\left\{f_{1},\ldots ,f_{M}\right\}\subset R^{\gamma \times \gamma }$ from *F* as support samples, where *F* represents the matrix to be binarized and *γ* represents the predefined sub-matrix.

Then, these sub-matrices are combined to obtain *M* × (*M* − 1) pairs of sub-matrices (*f*_*m*_, *f*_*m*′_), where *m*, *m*′ = 1, 2, …, *M* and *m* ≠ *m*′.

Consequently, by utilizing a kernel function $\kappa \left(f_{z},f_{z^{\prime }}\right):R^{\gamma \times \gamma }\times R^{\gamma \times \gamma }\mapsto R$, a set of hash functions mapping $H:R^{\gamma \times \gamma }\mapsto {\left\{1,-1\right\}}^{\lambda }$ is learned, where λ represents the number of hash functions. As a result, the multiplication operation between binarized values and precise floating-point values in the above equation can be replaced with simple addition. With this approach, the computational complexity is effectively reduced. In addition, the hash function mapping calculated by the following equation:

$$\begin{array}{c} \begin{array}{cc} H(Q_{d,h}) & =\text{sign}(\sum_{h=1}^{H}(\kappa (f_{m},F)-\mu_{h})A)=\text{sign}(g(f_{m})A)\\ & =[\begin{array}{ccc} h_{1}(f_{1}) & \ldots & h_{b}(f_{1})\\ \vdots & \ddots & \vdots \\ h_{1}(f_{M}) & \ldots & h_{1}(f_{M}) \end{array}]=[\begin{array}{c} H(f_{1})\\ \vdots \\ H(f_{M}) \end{array}] \end{array} \end{array}$$ (10)

where *κ*() represents the hash kernel function, $A\in R^{M\times b}$ denotes the weight matrix, the kernel function is normalized by $\mu_{i}=\frac{1}{M}\sum_{m=1}^{M}\kappa (f_{m},F)$ to ensure that it has a mean of 0, $g:R^{\gamma \times \gamma }$ is a mapping defined by $g\left(F\right)=\left[\kappa \left(f_{1},F\right)-\mu_{1},\ldots ,\kappa \left(f_{m},F\right)-\mu_{M}\right]\in R^{\gamma \times M}$.

**Fig 4:**
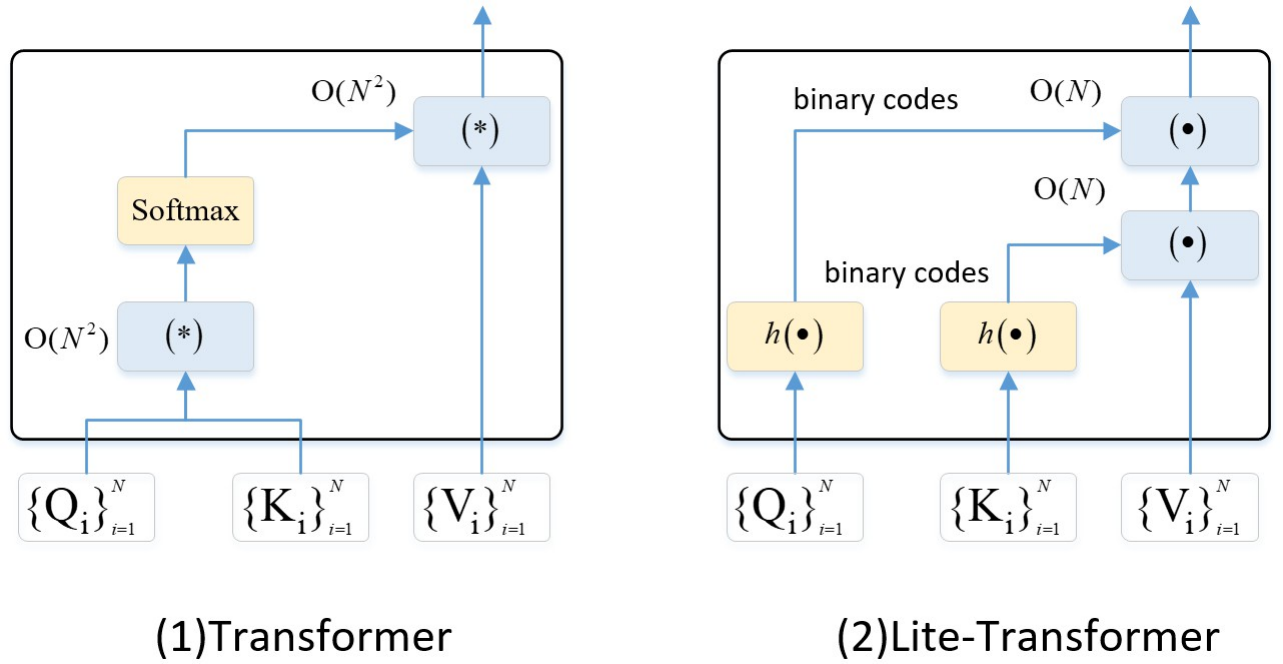
Computational graph for Lite-Transformer. With low-dimensional binary queries and keys, Lite-Transformer replaces the majority of energy-intensive floating point multiplications in the conventional Transformer with simple additions. This substitution results in a significant reduction in computational complexity.

As shown in Fig 4, By utilizing the mentioned binarization technique, the original high-order query *Q*_*d*,*h*_ and key *K*_*d*,*h*_ are transformed into a lower-dimensional space while still preserving their similarities. Consequently, this leads to a reduction in computational complexity.

Let (*q*_*d*,*h*_)_*m*_ represent the extracted submatrix of the query *Q*_*d*,*h*_. *H*((*q*_*d*,*h*_)_*m*_) denotes the vector after hash function mapping for query *Q*_*d*,*h*_. (*k*_*d*,*h*_)_*m*_ represents the submatrix obtained from key *K*_*d*,*h*_. *H*((*k*_*d*,*h*_)_*m*_) denotes the vector after hash function mapping for key *K*_*d*,*h*_.

For each submatrix pair (*q*_*d*,*h*_)_*m*_, (*k*_*d*,*h*_)_*m*′_), where *m*, *m*′ = 1, 2, …, *M*, the similarity measure is determined by the distance between their corresponding vectors *H*((*q*_*d*,*h*_)_*m*_) and *H*((*k*_*d*,*h*_)_*m*′_). The top *β* submatrix pairs with the highest similarity scores are selected to form the set $C_{q}$. On the other hand, the top *β* submatrix pairs with the lowest similarity scores are chosen to form the set $N_{q}$. Furthermore, the corresponding attention decay label *Y*_*d*,*h*_((*q*_*d*,*h*_)_*m*_, (*k*_*d*,*h*_)_*m*′_)) is calculated using the following equation:

$$\begin{array}{c} Y_{d,h}({\left(q_{d,h}\right)}_{m},{\left(k_{d,h}\right)}_{m^{\prime }}))=\{\begin{array}{cc} 1, & ({\left(q_{d,h}\right)}_{m},{\left(k_{d,h}\right)}_{m^{\prime }}))\in C_{q}\\ -1, & ({\left(q_{d,h}\right)}_{m},{\left(k_{d,h}\right)}_{m^{\prime }}))\in N_{q}\\ 0, & \;\text{otherwise}\; \end{array} \end{array}$$ (11)

As a result, an attention decay label matrix *Y*_*d*,*h*_ of size *M* × *M* is obtained. The student’s attention matrix *Head*_*h*_ is calculated by weighting the values *V*_*d*,*h*_ with the attention decay label *Y*_*d*,*h*_. The specific calculation expression is as follows:

$$\begin{array}{c} {Head}_{h}=\sum_{d=1}^{D}Y_{d,h}V_{d,h} \end{array}$$ (12)

The above formula can be interpreted as the student’s knowledge state being obtained by combining their answer records with the corresponding sub-spaces.

#### 3) Concatenation layer

In general circumstances, the Concatenation layer in GELT utilizes a multi-head attention mechanism to extract a knowledge state matrix denoted as *W*_*o*_, which has a size of *M* × *M*. This matrix is subsequently passed to the dot product module for further processing.

Within the Concatenation layer, the $Q_{d}\in R^{1\times M}$ corresponds to the question embedding vector *x*_*d*_ associated with the *d*- th historical answer record of the student.

$K_{d}\in R^{1\times M}$ represents the embedding vector*s*_*d*_, which denotes the average vector of the skill point embeddings involved in the *d*- th historical answer record. $V_{d}\in R^{1\times M}$ corresponds to the answer vector *e*_*d*_ for the *d*- th historical answer record. The answer vector *e*_*d*_ is obtained by concatenating the encoding vector of the question and the student’s answer indicator for that specific record.

#### 4) Fully connected layer

The fully connected layer is used to transform the multi-head attention matrix *Multi*_*Head*(*Q*, *K*, *V*) and generate a knowledge state matrix *W* with a size of *M* × *M*. Next, the dot product module calculates the inner product between the embedding vector $\hat{x}$ of the question to be predicted and the knowledge state matrix *W*, resulting in the matrix $\hat{X}=W\times \hat{x}$.

In order to enhance the generalization ability of the proposed architecture, the use of nonlinear activation functions is imperative. The sigmoid function is the most frequently used activation function in benchmark neural architectures due to its differentiability, which cause a continuous and differentiable output $\sigma (w\cdot \hat{X}+b)$ where *σ* is defined as:

$$\begin{array}{c} \sigma \left(\hat{X}\right)=\frac{1}{1+e^{-\hat{X}}} \end{array}$$ (13)

In this equation, *e* represents the mathematical constant Euler’s number (∼2.71828). To clarify further, the output of a activation neuron with vector ${\hat{x}}_{1}$ and corresponding weights *w*_*i*_, along with a bias term *b*.

$$\begin{array}{c} \sigma \left(\hat{X}\right)=\frac{1}{1+e^{-(\sum_{i}w_{i}{\hat{x}}_{i}+b)}} \end{array}$$ (14)

When the output of a nonlinear model is passed through the sigmoid activation function, it yields a value between 0 and 1. This value can be interpreted as a probability, denoted as $\hat{p}$, which represents the feasibility of the student correctly answering the new question. Typically, values above 0.5 indicate that the model predicts the positive class, while values less than 0.5 indicate the negative class.

### 3.4 The process of optimization

The training process of the proposed architecture involves optimizing the network parameters to minimize a cost function for the dataset. This optimization process is carried out using the back-propagation algorithm [38]. We have chosen the negative log-likelihood as the cost function:

$$\begin{array}{c} L=-\text{arg}\;\text{min}[{\tilde{p}}_{D+1}\;\text{ln}\;p_{D+1}+(1-{\tilde{p}}_{D+1})\;\text{ln}\;(1-p_{D+1})] \end{array}$$ (15)

where ${\tilde{p}}_{D+1}$ represents the true answer label, *p*_*t*+1_ represents the predicted answer identity based on the student’s cognitive state in time step *t* + 1. By training the parameters of the model, the objective is to reduce the error of cross-entropy, thereby ensuring that the model’s prediction gradually approximates the true labels.

## 4. Experiment and results

This section details the functioning of our strategies through the use of experimental demonstrations. Firstly, a concise introduction to the datasets used in our experiments will be provided. Next, we present the experimental protocol that we followed, along with a comparison against previous methods. Lastly, ablation studies are conducted on both the GELT and its prediction module to showcase their effectiveness in Section 4.5.

### 4.1 Datasets

In our experiment, we utilized three publicly available real-world datasets in the field of knowledge tracking: the ASSISTments2009 dataset [39], the ASSISTments 2012 dataset [40], and the EdNet dataset [41].

These datasets are widely recognized as popular temporal datasets that consist of interaction logs between students and real adaptive learning systems, such as Carnegie Learning’s Cognitive Tutor [42] and the ASSISTment system [40]. They offer extensive information about students, items, and their interactions, including start and end times, making them highly suitable for tracking changes in students’ knowledge over an extended period. The numbers of students, skills, questions, and question-response records are provided in Table 1.

**Table 1:** Datasets used for experiments.

Dataset	*ASSISTments2009*	*ASSISTments2012*	*EdNet*
Students	3,841	27,405	5,000
Skills	123	265	188
Questions	15,911	47,104	13,169
Records	190,320	1,867,167	222,141

#### The ASSISTments2009 dataset

This dataset has served as the benchmark for knowledge tracing studies over the last ten years. It comprises data from over 4,500 students across different subjects. For consistency, we adopted a similar data preprocessing approach as PEBG [14], which involved removing specific data, such as students with fewer than three records. Therefore, the ASSIST09 dataset includes 123 skills and encompasses data from 3,841 students who answered a total of 15,911 questions, resulting in 190,320 records.

#### The ASSISTments2012 dataset

An extended version of the ASSISTments2009 dataset, was collected from the same online intelligent tutoring system between 2012 and 2013. Following the same preprocessing method as before, the ASSIST12 dataset comprises 265 skills. It incorporates data from 27,405 students who answered a total of 47,104 questions, resulting in a comprehensive collection of 1,867,167 records.

#### EdNet dataset

The EdNet dataset released in 2020, is a comprehensive dataset that surpasses ASSISTments2009 and ASSISTments2012 in terms of recency. It was collected from the online educational platform EdNet.ai and encompasses more than 130 million student interactions. This dataset includes English content from diverse topics, including mathematics, science, and social studies. For these experiments, we utilized the EdNet-KT1 dataset, which consists of question-interaction documentation from students. We randomly selected 5,000 students from the source dataset, which contains 13,169 questions and 188 skills from 222,141 records.

### 4.2 Baseline methods

In order to demonstrate the effectiveness of our proposed architecture and showcase its improvements over existing deep learning based knowledge tracing models, we compared its predictive performance with the state-of-the-art deep KT model, including BKT, DKT and DKVMN.

#### BKT

The BKT model, initially introduced by Corbett and Anderson [5], is a widely utilized probabilistic model in knowledge tracing tasks. It operates under the assumption that the learning process can be represented as a series of distinct states: {*unlearned*, *learned*}, with each state denoting the mastery level of a particular skill or knowledge component.

#### DKT

The DKT [7] model integrates deep neural networks, which serves as a significant enhancement to traditional knowledge tracing models. Unlike BKT, which relies on handcrafted features and assumes discrete knowledge states, DKT employs recurrent neural networks to capture the temporal dependencies in a student’s interaction records. This allows DKT to enhance the accuracy and generalization performance of knowledge tracing.

#### DKVMN

In this model, a memory matrix is employed, consisting of key-value pairs that store information about the student’s interactions with the educational content. Each key-value pair correspondingly represents a specific knowledge component or skill. By leveraging this memory matrix, DKVMN [29] is able to effectively track and update the cognitive states of students based on their interactions over time.

### 4.3 Experimental setup

In the embedding propagation module of the graph neural network, we set the aggregate layer as *d* = {64, 128}, where the dimension of the vertex features is set to 64, and embedding size for skills and questions is set at 128. During the training phase, all embedding matrices are initialized randomly, and the parameters are continually updated throughout the training process.

The dataset size for knowledge tracing is relatively small, allowing our experiments to be conducted on a standard personal computer. In this experiment, our computing setup consists of a desktop computer equipped with an 8-core Intel Core i7-11800K processor, 32GB SDRAM, and an NVIDIA GeForce RTX 3060 GPU with 12288MB of memory. To align with the memory capacity of the GPU, a batch size of 128 was utilized during the GPU acceleration phase. In terms of the experimental setup, Python libraries, specifically TensorFlow [43] and Keras [44], were employed to implement and train the GELT model given their efficient functionalities. For model optimization and hyperparameter tuning, we leveraged the Adam algorithm [45] to optimize all trainable parameters. The initial learning rate was configured at 0.01, with a 10% decay scheduled every 10 epochs to prevent overfitting. Dropout regularization was implemented with a keep probability of 0.5 to enhance the model’s generalization capabilities. The experimental code can be referred to the link.

The weight coefficient *α* for the joint loss function in Eq (2) is initially set to 0.5. This setting ensures that the joint loss function carries the same weight as the other loss functions, resulting in a balanced impact on the model training.

In our experimental setup, we only included interaction records with a length greater than 3 for each sequence. Additionally, we divided 80% of all records for training and validation purposes, while allocating the remaining 20% as the test set.

Given the potential prediction skewness resulting from the imbalance between positive and negative samples in the dataset, solely assessing model superiority based on accuracy may lead to imprecise judgments. Therefore, in the KT field, the Area Under the Curve (AUC) is commonly utilized to evaluate model performance. The vertical axis of the AUC represents the True Positive Rate (TPR), while the horizontal axis represents the False Positive Rate (FPR), defined as follows:

$$\begin{array}{c} TPR=\frac{TP}{TP+FN} \end{array}$$ (16)

$$\begin{array}{c} FPR=\frac{FP}{FP+TN} \end{array}$$ (17)

Here, the TPR is calculated using True Positives (TP) and False Negatives (FN), indicating the ratio of positive samples correctly predicted by the model (also known as Recall). The False FPR is determined by False Positives (FP) and True Negatives (TN). Optimal model performance is achieved when TPR equals 1 and FPR equals 0. The closer the AUC is to the top-left corner, the better the classifier’s performance. The Area Under the Curve (AUC) represents the area beneath the ROC curve, offering robustness against sample class distribution, making it a reliable metric for evaluating classifier performance, particularly in handling imbalanced sample scenarios. Consequently, leveraging ROC curves and AUC for assessing and comparing model performance is a prevalent and recommended practice in the KT field.

To evaluate the effectiveness of various settings within the proposed framework, we conducted experiments using a 5-fold cross-validation approach. The key performance metric reported is the average test AUC derived from these cross-validation experiments.

### 4.4 Comparison of model

Table 2 showcases the performance comparison of GELT with state-of-the-art deep learning-based knowledge tracing methods on the three datasets. In the context of knowledge tracing methods, BKT serves as a representative traditional machine learning approach, embodying the probabilistic model utilized for estimating learners’ evolving knowledge states over time. DKT pioneered the integration of deep learning techniques, such as RNN and LSTM, to model students’ exercise processes and forecast their performance. Conversely, DKVMN leverages recurrent neural networks to monitor the mastery levels of individual knowledge concepts and construct learners’ knowledge states based on these proficiency levels. PEBG introduces a method that acquires pre-trained exercise embeddings and subsequently employs recurrent neural networks to project learners’ states, thus enhancing the precision of knowledge tracing. These diverse methodologies have broadened the research scope of knowledge tracing and facilitated more accurate predictions of learners’ knowledge states. From this comprehensive comparison, several noteworthy observations are as follows.

**Table 2:** The AUC results over three datasets.

Dataset	*ASSISTments2009*	*ASSISTments2012*	*EdNet*
BKT	0.647	0.615	0.562
DKT	0.735	0.701	0.690
DKVMN	0.739	0.675	0.689
PEBG	0.829	0.770	0.776
GELT	0.855	0.796	0.791
Gain%	3.01%	3.37%	1.81%

The proposed GELT model consistently achieved the highest AUC values across all three datasets. In particular, on the ASSISTments2009 dataset, GELT outperformed other deep learning-based methods, achieving an impressive AUC value of 0.855. This represents a significant gain of 3.01% on average compared to the AUC of 0.829 achieved by PEBG.

On the larger ASSISTments2012 dataset, GELT exhibited significant superiority over other competing methods, showcasing a performance improvement of 3.37% compared to the second-best performing method, as illustrated in Table 2. In general, GELT exhibits higher performance on larger datasets. This finding emphasizes the effectiveness of the graph structure in capturing relational features between the questions and the skills.

The GELT method, proposed for the EdNet dataset, exhibits a performance improvement of 1.81% in comparison to the PEBG approach. Of all the models compared, the BKT model shows the lowest performance. DKT and DKVMN both exhibit similar performance levels. Specifically, on the ASSISTments2009 dataset, GELT exhibits an improvement in the AUC by 11.5 and 11.9 compared to the closest baselines, such as DKT and DKVMN.

### 4.5 Ablation studies

To substantiate the key innovations in the GELT method, we conducted experiments to compare it with different model fusion methods. Specifically, we utilized pretrained embedding vectors as input for three deep models employed as classifiers: the DKT architecture leveraging recurrent neural networks, the Transformer model based on self-attention mechanisms, and our lite Transformer model.

Both accuracy (ACC) and Area Under the Curve were utilized as evaluation metrics to comprehensively assess the effectiveness of these innovations. Table 3 provides a comparison of evaluation metrics between our approach and the two classifiers on three publicly available datasets.

**Table 3:** The results of ablation experiments of three kinds of modules.

Dataset	*ASSISTments2009*	*ASSISTments2012*	*EdNet*
GE+DKT	0.829	0.769	0.768
GE+Transformer	0.854	0.794	0.791
GE+LiteTransformer	0.855	0.796	0791

Through our comparison of transformer architectures and recurrent neural networks, we observed that the two transformer architectures demonstrated enhanced flexibility, enabling them to effectively capture abundant information in large-scale datasets. This flexibility was further augmented by the utilization of self-attention mechanisms in transformer and lite-transformer methods. As a result, these two models are capable of capturing anterior-posterior dependent features of long time series information with greater accuracy.

**Fig 5:**
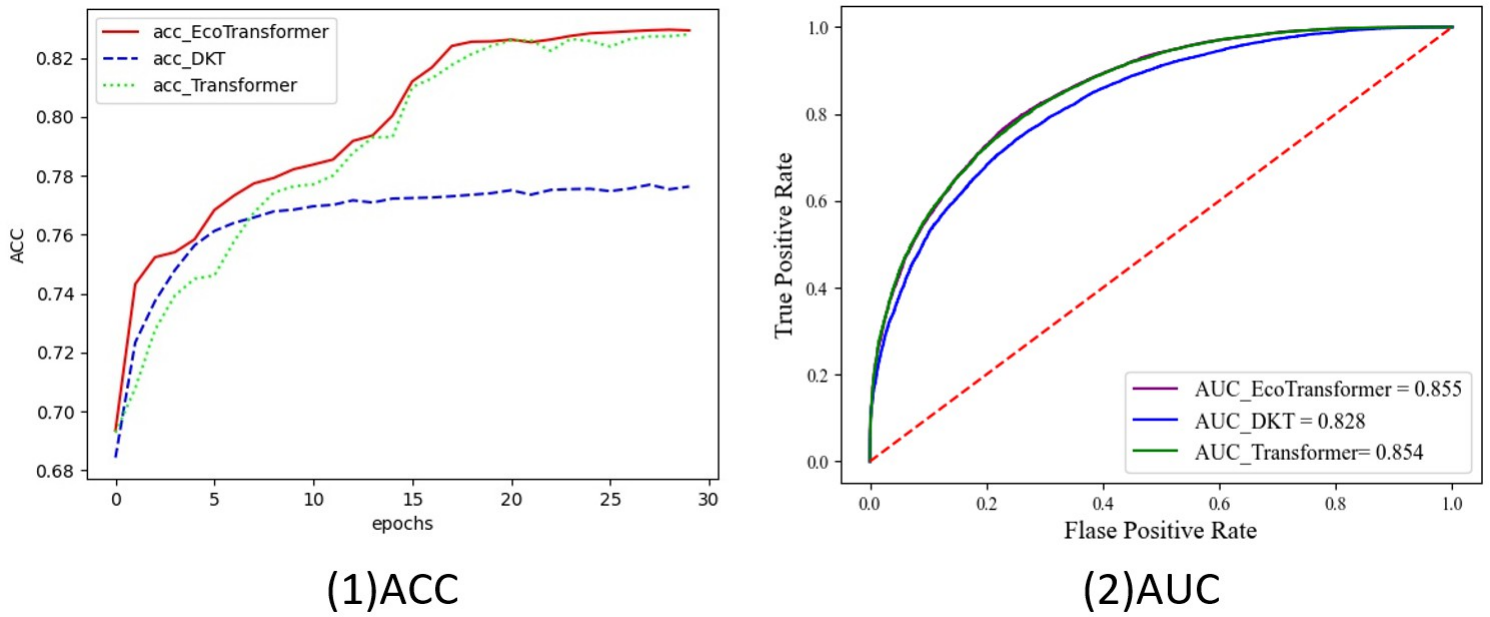
Comparison of ACC and AUC among three methods on the ASSISTments2009 dataset.

In Fig 5, the training curves of the GELT method and the comparison methods on the ASSISTments2009 dataset are illustrated. It is interesting to observe that the AUC curves of GELT and the Transformer method exhibit a significant overlap. However, it is worth noting that GELT consistently surpasses the DKT model in terms of both AUC and ACC values across all three publicly available datasets.

The experimental findings of this study highlight a significant improvement in the GELT model compared to the traditional attention mechanism. The GELT model exhibited an increase in the AUC value from 0.854 to 0.855 and an increase in the ACC value from 0.827 to 0.831. Although the utilization of the lite-transformer module resulted in only a slight improvement over the traditional transformer, subsequent experiments have shown that the lite-transformer can significantly reduce both training consumption and duration.

**Fig 6:**
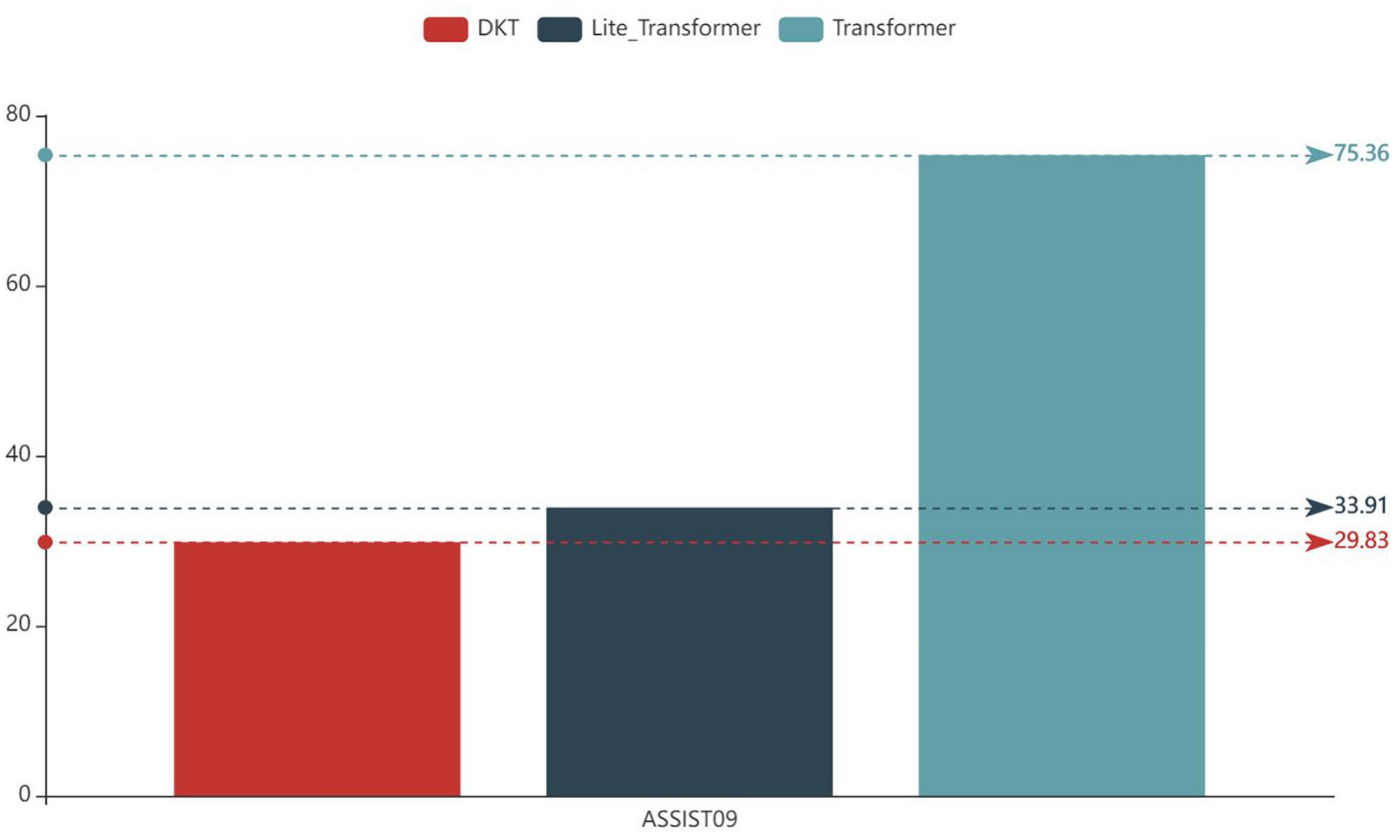
Comparison of training duration.

In Fig 6, a comparison of the computational costs and training duration is shown for GELT and other comparative methods on the ASSISTments2009 dataset. The results indicate that the DKT method requires approximately 29.83 seconds per epoch for training. On the other hand, the proposed method, which utilizes the conventional transformer, takes around 75.36 seconds per epoch. However, when the lite transformer is implemented, this duration decreases to 33.91 seconds per epoch.

This observation suggests that the Lite-Transformer offers an alternative to the computationally expensive floating-point multiplications found in the traditional Transformer. By employing low-dimensional binary queries and keys, the Lite-Transformer enables the use of simple additions instead. As a result, the Lite-Transformer significantly reduces the computational cost and training duration, making it a more efficient option for this task.

In conclusion, by incorporating the lite transformer, GELT manages to achieve a training cost that is on par with the classical DKT method. Additionally, GELT maintains a high level of prediction accuracy while significantly reducing computational costs when compared to using the conventional attention mechanism. This highlights the effectiveness of the lite transformer in optimizing the computational efficiency of the model without sacrificing its predictive capabilities.

### 4.6 Visualization of interpretability

**Fig 7:**
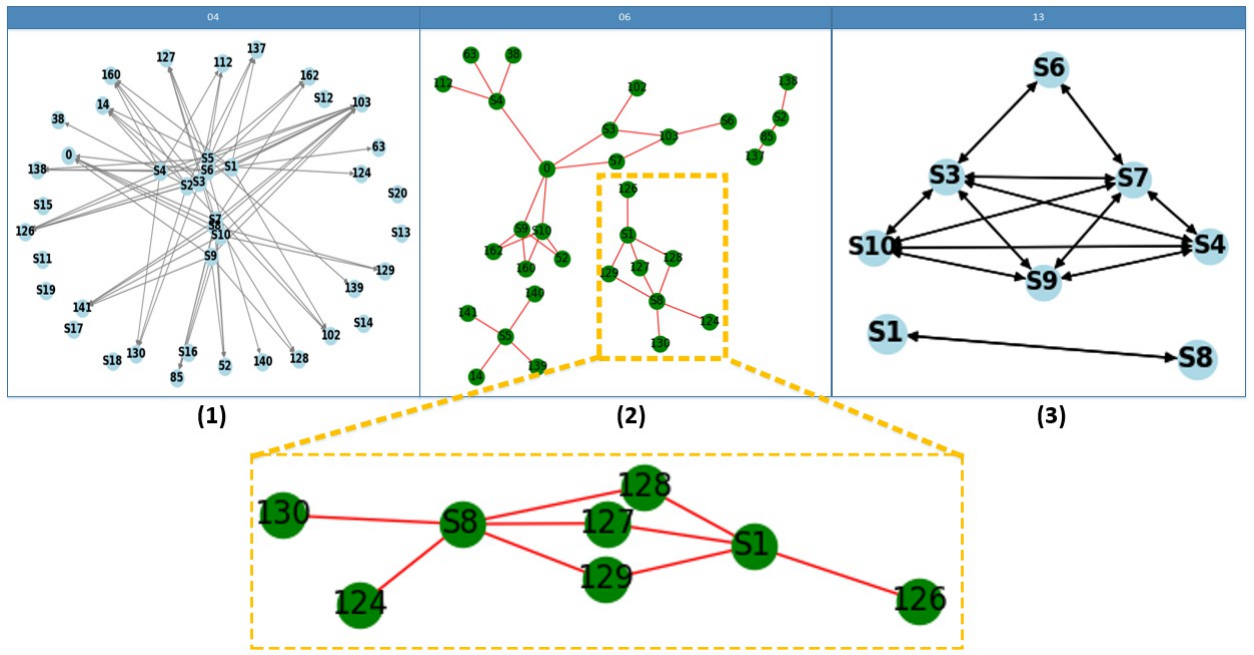
The graph illustrates the correlation between skills, which is determined by the weights learned from associating the same questions. By examining the graph, we can observe that it uncovers the implicit relationships between skill nodes, leading to a clear clustering effect.

To demonstrate the interpretability of the GELT model, as shown in Fig 7, we utilize graph embeddings to visually depict the interconnected relationship among skills in the ASSISTments2009 dataset.

Firstly, a random selection of 10 skills from the dataset was performed, including the associated questions for each skill point. Each question node was then visited, and the corresponding skills were connected using edges. It is crucial to note that there is a many-to-many relationship between questions and skills. As depicted in Fig 7-1, there is no direct association between skills before graph embedding.

Fig 7-2 illustrates a graph structure in which skills and questions are connected. Some questions are linked to specific skills, while a question can be associated with multiple skills. However, the relationship between skills is implicitly established through the indirect association of “sharing a common question.”

An intriguing observation is that the GELT model enables the discovery of the incidence relation between two skills that belong to the same question. This phenomenon is demonstrated in Fig 7-3, where skill points S8 and S1 share questions with the sequence numbers 127-129. After pretraining the graph embedding, the association weight between S1 and S8 increases, indicating a direct association between these two skills. In contrast, skill S2 is associated with questions 85 and 138, while skill S5 is associated with questions 14, 139, 140, and 141. However, these questions are not connected to other skills. Consequently, after encoding, S2 and S5 become isolated skills, respectively.

**Fig 8:**
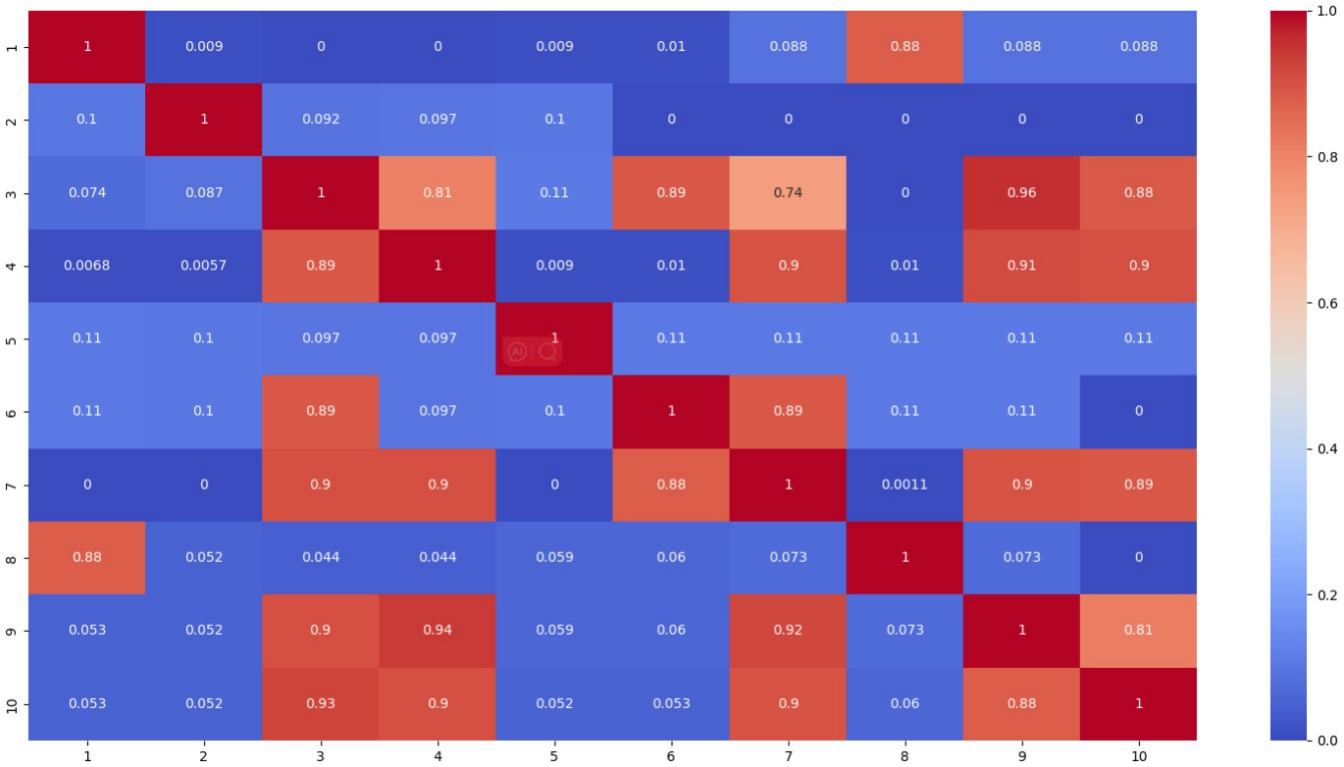
The heatmap representing the weighted values that indicate the relationships between different skills.

To represent the interplay between skill tags, Fig 8 illustrates a heatmap that corresponds to the relevance matrix of skills in the given graph. The analysis is based on the ASSISTments2009 dataset, which includes question sequences with skill numbers ranging from 1 to 123.

Initially, there is no direct association between skills, leading to weight values close to zero, indicating a low similarity between them. However, as the graph is aggregated and embedded, there are changes observed in the weight values for certain skills.

One notable finding from Fig 8 is the diagonal, which represents the correlation of each skill with itself. In this case, skills S2 and S5 have weight values close to zero as they are not associated with any other skills. Conversely, skills S8 and S1 show a strong correlation (weight value close to 1) because they are both connected to questions 127-129. This correlation implies that achieving mastery in either skill S3 or S6 indicates successful performance in answering questions 127-129.

This finding implies that learners update their knowledge state after answering a question, reflecting their mastery of specific knowledge. The GELT model effectively evaluates learners’ skill proficiency by accurately predicting their likelihood of answering questions correctly. The prediction approach of this model aligns closely with how humans model knowledge states. It incorporates learners’ dynamic learning process, integrates question correctness and skill status, and evaluates exercise difficulty to assess their knowledge state. Consequently, the model’s predictive ability is enhanced.

## 5. Conclusion and future work

In this paper, we introduce a novel approach to knowledge tracing that combines graph embedding techniques with a Lite Transformer model. The proposed method utilizes a graphical model to capture the relationships between problem-skill, problem-problem, and skill-skill. This graph-based representation is further enhanced through a pre-training module, which aggregates high-level embeddings. Finally, the obtained embedding vectors are input into the Lite Transformer module to predict the student’s knowledge state.

The integration of graph embedding and the Lite Transformer addresses the challenge of knowledge tracing by considering both the dependencies between problems and skills and the importance of different skill concepts. The graphical model provides a more comprehensive representation of the underlying relationships, while the Lite Transformer module enables accurate predictions of the student’s knowledge state. Experiments conducted on three real-world datasets demonstrate that our proposed model achieves comparable performance to conventional attention models while consuming significantly fewer resources. By combining graph embedding techniques with the Lite Transformer, this approach offers a promising framework for knowledge tracing in education, with potential applications in personalized learning systems.

There are several potential directions for future work:

1. In personalized learning, offering interpretable feedback and actionable recommendations is crucial in assisting learners to achieve enhanced learning outcomes. For the task of knowledge tracing, integrating interactive and explainable techniques can offer better insight into predictions. One potential method to enhance interpretability is to employ effective attention mechanisms to capture detailed information at the exercise level and learners’ prior knowledge. This integration can enable users to better understand the model’s predictions and enhance the transparency of the knowledge tracing process overall.
2. Reinforcement learning presents a powerful framework for knowledge tracing tasks, offering the potential to significantly enhance the personalized learning experience for students. Further exploration and application of reinforcement learning algorithms can lead to improved adaptive learning systems. Investigating how reinforcement learning can be combined with existing knowledge tracing models can help create more effective and dynamic learning environments tailored to individual students’ needs.
